# Lateralization of ERPs to speech and handedness in the early development of Autism Spectrum Disorder

**DOI:** 10.1186/s11689-017-9185-x

**Published:** 2017-02-05

**Authors:** Kayla H. Finch, Anne M. Seery, Meagan R. Talbott, Charles A. Nelson, Helen Tager-Flusberg

**Affiliations:** 1 Department of Psychological and Brain Sciences, Boston University, 64 Cummington Mall, Boston, MA 02215 USA; 2 New York University School of Medicine/Bellevue Hospital Center, 462 First Avenue, New York, NY 10016 USA; 3 MIND Institute, University of California Davis, 2825 50th Street, Sacramento, CA 95817 USA; 4 Laboratories of Cognitive Neuroscience, Division of Developmental Medicine, Boston Children’s Hospital, 1 Autumn Street, Boston, MA 02215 USA; 5 Harvard Medical School, 25 Shattuck Street, Boston, MA 02215 USA; 6 Harvard Graduate School of Education, 13 Appian Way, Cambridge, MA 02138 USA

**Keywords:** Autism spectrum disorders, Event-related potentials, Speech processing, Lateralization, Handedness

## Abstract

**Background:**

Language is a highly lateralized function, with typically developing individuals showing left hemispheric specialization. Individuals with autism spectrum disorder (ASD) often show reduced or reversed hemispheric lateralization in response to language. However, it is unclear when this difference emerges and whether or not it can serve as an early ASD biomarker. Additionally, atypical language lateralization is not specific to ASD as it is also seen more frequently in individuals with mixed- and left-handedness. Here, we examined early asymmetry patterns measured through neural responses to speech sounds at 12 months and behavioral observations of handedness at 36 months in children with and without ASD.

**Methods:**

Three different groups of children participated in the study: low-risk controls (LRC), high risk for ASD (HRA; infants with older sibling with ASD) without ASD, and HRA infants who later receive a diagnosis of ASD (ASD). Event-related potentials (ERPs) to speech sounds were recorded at 12 months. Utilizing a novel observational approach, handedness was measured by hand preference on a variety of behaviors at 36 months.

**Results:**

At 12 months, lateralization patterns of ERPs to speech stimuli differed across the groups with the ASD group showing reversed lateralization compared to the LRC group. At 36 months, factor analysis of behavioral observations of hand preferences indicated a one-factor model with medium to high factor loadings. A composite handedness score was derived; no group differences were observed. There was no association between lateralization to speech at 12 months and handedness at 36 months in the LRC and HRA groups. However, children with ASD did show an association such that infants with lateralization patterns more similar to the LRC group at 12 months were stronger right-handers at 36 months.

**Conclusions:**

These results highlight early developmental patterns that might be specific to ASD, including a potential early biomarker of reversed lateralization to speech stimuli at 12 months, and a relation between behavioral and neural asymmetries. Future investigations of early asymmetry patterns, especially atypical hemispheric specialization, may be informative in the early identification of ASD.

## Background

Autism spectrum disorder (ASD) is a neurodevelopmental disorder characterized by deficits in social communication and presence of repetitive and restricted behaviors [1]. Although there is a general consensus that ASD originates early in life from predominately biological and genetic origins, these behavioral symptoms do not emerge until the second year of life, and a clinical diagnosis is often not received before the third birthday [2]. To help detect atypicalities that arise prior to diagnosable behaviors, researchers have turned towards studying younger siblings of children with ASD [3, 4]. These siblings are considered high risk for developing ASD (HRA) with approximately 20% later receiving a diagnosis of ASD [5, 6] compared to only 1.5% in the general population [7]. Past research has found that many HRA infants who later received an ASD diagnosis display behavioral symptoms within the first 2 years of life (see [3, 8, 9] for reviews). However, subtle atypicalities, including social impairments and delays in verbal and gestural communication, have also been found as early as 12 months in HRA infants who do not go on to develop ASD [10–13]. These subtle atypicalities, or endophenotypes, are heritable biomarkers seen in both affected and unaffected family members [14, 15]. When studying the early development of ASD, it can be difficult to separate endophenotypes from early biomarkers of risk for ASD. Early biomarkers of risk for ASD are specific to the HRA infants who later receive a diagnosis of ASD and are not seen in the unaffected HRA infants. Despite the difficulties, researchers are starting to identify early behavioral differences in HRA infants who later received a diagnosis of ASD, including differences in looking behaviors towards faces and social scenes [16–18].

While identifying early behavioral atypicalities is valuable, use of functional brain measures, such as event-related potentials (ERPs) and electroencephalogram (EEG), might offer more insight as these measures uncover infants’ perception and processing abilities without being restricted to infants’ behavioral capabilities. Indeed, several possible neural ASD risk markers and endophenotypes have been proposed, primarily within the social domain. For instance, in HRA infants, atypical EEG patterns and ERPs have been found in response to direct eye gaze [19], faces and objects [20, 21], and familiar and unfamiliar faces [22, 23]. Additionally, initial evidence from functional near-infrared spectroscopy show that HRA infants exhibit atypical neural processing to dynamic social stimuli [24, 25]. Finally, atypical trajectories of EEG power [26, 27] and neural connectivity [28, 29] have also been found in HRA infants. Most research looking into neural development of HRA infants looks at the group as a whole, investigating all infants at familial risk. However, there is some promising evidence in identifying neural biomarkers of risk for ASD including distinct patterns in neural connectivity [28, 29] and lateralization in response to faces [20] in the subset of HRA infants who go on to develop ASD.

Past research has mostly focused on neural response to social stimuli, but fewer studies have looked into those related to language processing. Language ability, while highly heterogeneous and no longer a core symptom of ASD as outlined by the most recent DSM-5 [1], is often impaired in individuals with ASD with many displaying milestone delays and impairments in their vocabulary development [30–32]. Even children and toddlers with ASD and normal language abilities show subtle atypicalities in their language processing [33–35]. Language is a highly lateralized function with left hemispheric dominance emerging as early as a few days after birth in the majority of typically developing individuals [36, 37]. Conversely, in toddlers, children, and adults with ASD, numerous studies have demonstrated reduced or reversed hemispheric lateralization of both structure and function for neural regions associated with language (see [38, 39] for reviews). In addition, in an earlier study from our research program, we found atypical lateralization patterns of ERPs in response to speech sounds in HRA infants as young as 12 months of age [40]. These HRA infants exhibited reversed lateralization compared to low-risk control (LRC) infants in a later negative-going slow wave (LSW) observed over the anterior regions from 300 to 700 ms after the presentation of speech sounds. However, it is unknown whether the reversed lateralization response in the HRA infants was being driven by those who later received a diagnosis of ASD. The current study expands on Seery and colleagues’ [40] sample to examine differences in lateralization patterns in response to speech between infants who go on to develop ASD and infants who do not determine if early atypical lateralization patterns are an endophenotype or a biomarker of risk for ASD.

Atypical lateralization to language is not specific to ASD. Reduced or reversed language lateralization is also seen more frequently in typically developing children and adults with mixed- and left-handedness [41–43], though few studies have extended this investigation to include individuals with ASD. Using functional neuroimaging, Knaus and colleagues [44] found that adolescents with ASD who showed more typical left language asymmetry were less likely to be left-handed. However, it is still unclear how this relation manifests in early development of ASD and HRA infants.

In typically developing populations, a right-handed bias may emerge as early as 15 weeks of gestation. Approximately 90% of fetuses show a strong right-handed preference for thumb-sucking [45], and this preference continues postnatally as infants are more likely to reach and grasp with their right hand [46]. Despite these early biases, handedness is malleable into early childhood [47]. Because of this, there is no consensus on when adult-like handedness is acquired. However, some researchers have argued that the direction of handedness is fixed by the age of 3 and then continues to strengthen through late childhood (see [48] for a review). Despite the malleability in infancy and toddlerhood, there does seem to be a relation between early hand preferences and language abilities [49–51] with increased rates of left- and mixed-handedness in children with language difficulties [52] and developmental disorders that often have language impairments including dyslexia [53] and ASD [54–57].

Given the increased prevalence of left- and mixed-handedness in ASD, it is important to consider its development when looking at differences in lateralization patterns of ASD. Atypical lateralization patterns and higher incidences of left- and mixed-handedness might represent a single underlying biomarker of risk for ASD, such as atypical cortical specialization. By exploring the early development of hand preferences, we would better understand the relation between these two domains. The purpose of this study was to examine differences in lateralization to speech sounds in infancy as well as the relation between early lateralization patterns and later hand preferences in toddlerhood. First, we expand on the findings of Seery et al. [40] by examining differences in lateralization of the ERPs to speech sounds at 12 months in LRC and HRA infants. We were specifically interested in whether infants who later develop ASD differed in the lateralization of their ERP responses compared to the LRC and HRA infants. We hypothesized that infants who received a diagnosis of ASD would show reversed lateralization patterns compared to LRC infants, and HRA infants without ASD would show an intermediate, dampened lateralization pattern. We also measured handedness in these infants at 36 months utilizing a novel observational approach. We hypothesized that three-year-old children with ASD will show more left- and mixed-handedness tendencies compared to their typically developing peers, who will begin to show a stronger right-hand preference. Finally, we investigated the relations between early lateralization patterns to speech, later handedness patterns of behavior, and language and cognitive abilities. We hypothesized that across all groups, infants who show more typical lateralization patterns at 12 months will show stronger right-handedness at 36 months. We also predicted that differences within these patterns would relate to the child’s cognitive abilities, specifically language.

## Methods

### Participants

Participants were infants enrolled in an IRB-approved collaborative longitudinal study conducted at Boston Children’s Hospital/Harvard Medical School and Boston University. Families were excluded from the study based on child gestational age of less than 36 weeks, time spent in neonatal intensive care, maternal steroid use during pregnancy, maternal diabetes, family history of genetic disorders, non-English-speaking households (English spoken less than 75% of the time), or exposure to a language that uses the phonemic contrast investigated (e.g., Bengali or Hindi). Infants were enrolled in one of two groups: low-risk control (LRC) which included infants with no family history of ASD, and high-risk for ASD (HRA), which included infants with an older siblings diagnosed with ASD. All older siblings had a community diagnosis of ASD, and in most cases, their diagnoses were confirmed independently in the project with the Social Communication Questionnaire [58] or Autism Diagnostic Observation Schedule (ADOS; [59]). Once enrolled, infants completed visits from 3 to 36 months of age. Informed consent was obtained at the family’s first visit. Children who completed either the 12- or 36-month visit were considered for inclusion in the current study. A total of 164 children completed the behavioral assessments at 36 months and were used to create the handedness measure. Of these, 151 children received a final diagnosis including 69 LRC, 60 HRA who did not go on to develop ASD (HRA), and 22 children who received a diagnosis of ASD (ASD). At 12 months, 96 infants (44 LRC, 39 HRA, 13 ASD) contributed usable ERP data, of which, 61 were included in Seery et al. [40]. Of the 96 infants, 84 (40 LRC, 32 HRA, 12 ASD) also contributed handedness data at 36 months. Detailed behavioral profiles for these groups at children at time points from 12 to 36 months are given in Table 1.

**Table 1:** Behavioral characteristics of participants included in analyses

	Group			
	LRC	HRA	ASD	LRC vs. HRA vs. ASD^a^
Total *N*	73	67	23	
Male/female	35:38	32:35	16:7	
ADOS severity scores				
18 months (*N*)	65	62	19	
Mean (SD)	1.40 (0.9)	2.11 (1.7)	3.42 (2.7)	***
24 months (*N*)	69	65	21	
Mean (SD)	1.64 (0.8)	2.09 (1.4)	5.24 (2.4)	***
36 months (*N*)	68	58	20	
Mean (SD)	1.43 (1.2)	1.55 (1.1)	5.45 (1.6)	***
MSEL *T* scores				
12 months (*N*)	73	66	22	
Fine Motor	59.60 (8.9)	60.64 (9.5)	57.77 (10.5)	n.s.
Visual Reception	56.88 (8.8)	54.68 (8.7)	51.14 (7.0)	*
Receptive Language	45.29 (7.6)	44.74 (8.2)	40.18 (8.6)	*
Expressive Language	49.79(8.5)	46.14 (7.8)	44.05 (11.0)	**
18 months (*N*)	67	64	19	
Fine Motor	52.72 (6.7)	53.25 (7.6)	48.68 (6.7)	*
Visual Reception	52.12 (8.7)	50.39 (7.7)	44.62 (10.0)	**
Receptive Language	54.15 (14.1)	47.08 (14.6)	41.42 (17.7)	**
Expressive Language	50.43 (6.8)	49.77 (11.0)	45.32 (12.2)	n.s.
24 months (*N*)	71	61	23	
Fine Motor	53.75 (10.6)	51.41 (8.4)	47.09 (10.3)	*
Visual Reception	56.75 (10.9)	54.92 (9.5)	49.83 (12.0)	*
Receptive Language	58.82 (7.8)	54.55 (8.9)	46.09 (17.0)	***
Expressive Language	56.65 (9.8)	53.41 (9.9)	49.78 (12.8)	**
36 months (*N*)	68	55	18	
Fine Motor	55.16 (14.1)	49.18 (13.5)	40.53 (10.5)	***
Visual Reception	62.66 (11.1)	57.40 (10.9)	51.24 (12.1)	***
Receptive Language	57.87 (8.1)	54.02 (10.1)	46.11 (11.6)	***
Expressive Language	60.94 (7.1)	55.47 (8.9)	48.22 (9.1)	***

^a^One-way ANOVAs for comparisons of MSEL *T* test scores, Kruskal-Wallis tests for comparison of ADOS severity scores

*** <0.001, ** < 0.01, * < 0.05

ASD diagnoses for the participants were based on the ADOS [59] administered at 24 or 36 months in addition to expert clinical judgment at 36 months. The ADOS is a semi-structured, standardized assessment that consists of social and play activities to elicit behaviors related to diagnosis of ASD. A severity score (range: 1–10) is obtained through the ADOS with a score of 4 or higher indicative of ASD. The ADOS was administered by research staff with extensive experience in testing children with developmental disorders and co-scored by an ADOS-reliable researcher via video recording. Cases of concern (those meeting criteria on the ADOS or coming within 3 points of cutoff) were reviewed by a licensed clinical psychologist who evaluated video recordings of behavioral assessments along with the scores from those assessments to determine final clinical judgment: no clinical concern/typically developing, ASD, or non-spectrum concerns (e.g., ADHD, anxiety, language delay). Infants were included in the ASD group if they had a severity score of 4 or higher on the ADOS at 36 months and received a final clinical judgment of ASD at 36 months. For two participants, 36-month ADOS scores and/or clinical judgment were not available, but they were included in the ASD group because they received a score of 4 or higher on the ADOS and a diagnosis of ASD by a clinician at 24 months.

Because language impairments are more common amongst HRA and ASD children, analyses evaluating group differences in ERPs to speech are presented both with and without infants who experience any difficulty with language during toddlerhood. Language difficulty was determined from the Mullen Scales of Early Learning (MSEL; [60]), a developmental assessment with Fine Motor, Visual Reception, Expressive Language, and Receptive Language subscales. Infants were considered to have language difficulties if they had a standardized *T* score lower than 30 on either the Expressive Language or Receptive Language subscale at 18, 24, or 36 months or *T* scores lower than 35 on both language subscales at a single age. At 12 months, 66 infants (36 LRC, 26 HRA, 4 ASD) had completed the MSEL at all of these time points and did not later meet criteria for language difficulties.

### 12-month visit: ERPs to speech stimuli

#### Stimuli

At 12 months, infants listened to a stream of three different consonant-vowel stimuli that were presented in a random order using a double-oddball procedure (following [61]). A standard (voiced, unaspirated, retroflex stop;/ɖa/) was presented 80% of the time, a native deviant (voiceless, aspirated retroflex palatal stop;/ta/) was presented 10% of the time, and a non-native deviant (a voiced, unaspirated dental stop;/da/) was presented the remaining 10% of the time. This double-oddball paradigm was originally used to measure the development of perceptual narrowing as reported in Seery et al. [40], but it is not the focus of the analyses in this paper. The three conditions were kept separate as a repeated factor variable, but we did not predict any differences across the speech percepts in the LSW.

#### Procedure

ERPs were recorded as infants sat on a parent’s lap in an electrical- and sound-shielded testing room with low lighting. A maximum of 600 stimuli were presented with varying interstimulus intervals via two bilateral loudspeakers at 80 dB. An experimenter was in the room and blew bubbles throughout the procedure to maintain the infants’ interest and increase toleration of the electrode net. The procedure took approximately 15 min.

#### Analysis of electrophysiological data

Continuous EEG was recorded using either a 64-channel Geodesic Sensor Net or a 128-channel HydroCel Geodesic Sensor Net (Electrical Geodesics Inc., Eugene, OR) referenced online to vertex (Cz). The electrical signal was amplified with either a NetAmps 200 or NetAmps 300 amplifier (Electrical Geodesics Inc.)^1^ using a 0.1–to 100-Hz band-pass filter, digitized at 250 Hz, and stored on a computer drive before being processed offline using NetStation 4.5.1 analysis software (Electrical Geodesics Inc.; see Table 2 for details of equipment used for each participant). EEG was segmented into 700 ms post-stimulus epochs with a baseline period of 100 ms. The segments were then digitally filtered using a 30-Hz low-pass elliptical filter and baseline-corrected using mean voltage during the 100 ms pre-stimulus baseline period.

**Table 2:** Participant and equipment details at the 12-month ERP visit

	Group			
	LRC	HRA	ASD	LRC vs. HRA vs. ASD^a^
*N*	44	39	13	
Age days (SD)	373.18 (9.1)	375.03 (8.9)	376.00 (14.3)	n.s.
Male/female	5:6	6:7	6:7	
Geodesic Sensor Net: Hydrocel Sensor Net	7:15	22:17	4:9	
NetAmp 200: NetAmp300	23:21	31:38	8:5	
Number of standard trials (SD)	31.70 (8.8)	29.69 (10.7)	35.46 (11.1)	n.s.
Number of non-native deviant trials (SD)	30.11 (7.9)	28.05 (10.7)	33.46 (11.2)	n.s.
Number of deviant trials (SD)	29.55 (8.2)	27.90 (10.7)	32.31 (9.2)	n.s.

^a^One-way ANOVAs for comparisons of age and number of trials for each condition

n.s. >0.05

In line with past research that uses the double-oddball procedure [40, 61], three categories of segments were created: standard (containing only standard trials that immediately followed a deviant), native deviant, and non-native deviant. Average waveforms for the speech percept were re-referenced to the average reference. Segments were visually examined by an experimenter blind to the study group. Individual channels were marked bad if contaminated by artifacts including body movement, eye movement, eye blinks, or off-scale activity (±200 μV). If more than 15% of the channels in a single segment were marked as bad, the whole segment was excluded from further analysis. Participants with fewer than 10 good trials in any of the conditions were excluded from remaining analyses. For all remaining participants, bad channels of accepted segments were replaced using spherical spline interpolation and average waveforms for each individual participant was generated and re-referenced to the average reference. For the final sample, the number of trials did not differ across groups within any of the conditions (all *p* > 0.20; see Table 2).

**Fig. 1:**
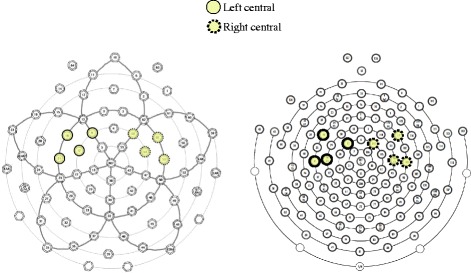
Electrode groupings used for the 64-channel Geodesic Sensor Net (*left*) and 128-channel HydroCel Sensor Net (*right*)

As in our previous work [40], a later negative-going slow wave (LSW) was observed over the anterior regions over the second half of the epoch (300–700 ms). Two regions of interest were constructed from anterior electrodes: left central (FC1, FC5, C3, C5) and right central (FC2, FC6, C4, C6; see Fig. 1 for details on the selected electrodes).

### 36-month visit: handedness measure

Hand preference was measured on a variety of behavioral observations during the ADOS and MSEL for all 164 children who completed the behavioral assessments at 36 months. The fine motor scale of the MSEL and the social communicative play of the ADOS give rise to multiple behaviors that often show dominant hand tendencies. Also, given the nature of the ADOS and MSEL, the hand of the child chooses to use for these behaviors is typically not prompted and thus is likely to reflect the child’s preference. Behaviors were selected because of their similarity with items on the Edinburgh Handedness Inventory (EHI; [62]) and included seven items: hand used to draw, complete a picture-maze, cut with a knife, use a fork/spoon, cut with scissors, use a toothbrush, and throw a ball. Each behavior was scored on a 5-point Likert scale: always left, usually left, no preference, usually right, and always right. 20% of the behaviors were double-coded and using Cohen’s ĸ, high interrater reliability was observed for all behaviors (ĸ > 0.79, *p* < 0.001)

### Statistical approach

#### ERPs at 12 months

We computed a 3×2×3 ANOVA with condition (standard, non-native deviant, native deviant) and hemisphere (left, right) as within-subjects factors and outcome group (LRC, HRA, ASD) as a between-subjects factor, using the mean amplitude of the LSW as the dependent variable. Significant interaction effects were explored further using reduced ANOVAs. To account for multiple comparisons, Bonferroni corrections were applied.

#### Handedness at 36 months

Confirmatory factor analysis (CFA) was performed on a theory-driven model that a univariate latent measure (i.e., handedness) underlay the individual behavioral measures. CFA was completed using Mplus, Version 7 [63]. Given that some have argued that 5-point Likert scales should be treated as a continuous variable, while others suggest it is an ordinal variable, analyses were conducted using both robust mean and variance adjusted weighted least squares (WLSMV) and robust maximum likelihood (MLR) estimator methods [64, 65]. WLSMV treats the variables as categorical while making no distribution assumptions and does not require a large sample. On the other hand, MLR assumes the data to be continuously distributed but allows for missing data and variation from normal distribution [65]. Applying the principals outlined by Brown [66], overall model fit was assessed by several fit indices including chi-square statistics and fit indices provided by the Mplus output including the root mean square error of approximation (RMSEA), comparative fit index (CFI), Tucker-Lewis index (TLI), and the weighted root mean square residual (WRMR) for WLSMV estimation and standardized root mean square residual (SRMR) for MLR estimation.

Based on the CFA, we used the relevant behavioral measures to compute a composite score similar to what is calculated on other handedness measures (e.g., the EHI). This was computed by subtracting the sum of tasks the child preferred to use his/her left hand from the sum of the tasks the child preferred using his/her right hand, dividing the difference by the cumulative sum of the hand tasks, and multiplying this by 100 ([RH-LH]/[RH + LH]*100). The composite score ranged from −100 to +100 with negative values indicating a preference for the left hand, a 0 indicating no hand preference, and positive values indicating a preference for the right hand. We compared group differences for the children in this study in the composite score using the Kruskal-Wallis H test.

#### Associations between lateralization, handedness, and behavioral measures

We created a laterality index based on the central sites for the LSW by subtracting the mean amplitude of the LSW over the right central sites from the mean amplitude over the left central sites (LSW_left_–LSW_right_). We computed Spearman’s rho correlations between the LSW laterality index at 12 months, the composite handedness score at 36 months, and the four subscales of the MSEL (Fine Motor, Visual Reception, Expressive Language, and Receptive Language) at respective time points. We investigated each of these relations across all the participants as well as within the three groups (LRC, HRA, ASD). Bonferroni’s correction for multiple comparisons was applied.

## Results

### ERPs at 12 months: full sample

**Fig. 2:**
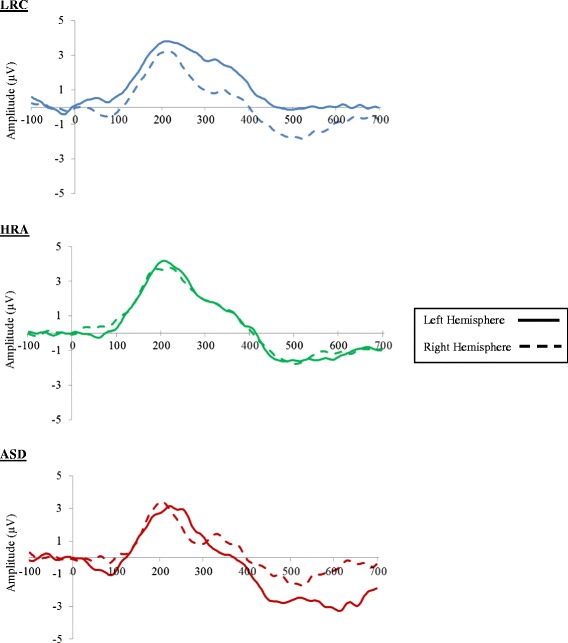
Grand-averaged waveforms across all conditions over *left*- and *right-hemisphere* central electrode sites at 12 months

Waveform graphs of the LSW laterality index over the central electrodes are shown in Fig. 2.

**Fig. 3:**
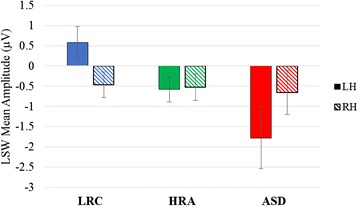
Average LSW for each hemisphere across all conditions at 12 months

The ANOVA revealed a significant hemisphere by group interaction effect (F(2,88) = 4.15, *p* = 0.019; see Fig. 3). No other main or interaction effects were observed (all *p* > 0.1). Given that there were no main or interaction effects related to condition, we created mean amplitudes of LSW for each hemisphere by collapsing across conditions. Follow-up analyses for the hemisphere by group condition revealed no group differences in the right hemisphere (*p* = .956) and significant group differences in the left hemisphere (*p* = .005). Specifically, LRC infants were significantly different from ASD infants (*p* = 0.007) with LRC infants having a more positive LSW mean amplitude over the left hemisphere (mean = 0.57 SD = 2.65) compared to ASD infants (mean = −1.78, SD = 2.73). HRA infants (mean = −0.58, SD 1.93) did not significantly differ from the LRC (*p* = 0.092) or ASD infants (*p* = 0.361).

### ERPs at 12 months: no language difficulties

The ANOVA with the reduced sample (excluding those with lower language scores) showed similar results revealing a significant hemisphere by group interaction (F(2,63) = 3.33, *p* = 0.042). No other main or interaction effects were observed (all *p* > .08). Again, given that there were no main or interaction effects related to condition, follow up analyses used mean amplitude of LSW for each hemisphere, collapsing across conditions. Follow up analyses revealed no group differences in the right hemisphere (*p* = 0.850) and a marginally significant group difference in the left hemisphere (*p* = 0.051). Specifically, LRC infants had a more positive LSW mean amplitude over the left hemisphere (mean = 0.65, SD = 2.66) compared to ASD infants (mean = −2.10, SD = 3.34). HRA infants (mean = −0.48, SD = 2.08) did not significantly differ from the LRC (*p* = 0.250) or ASD infants (*p* = 0.688).

### Handedness at 36 months

As seen in Table 3, CFA analysis using all seven items indicated good fit for both estimation methods. Medium to high factor loadings were observed with parameter estimates for the 7-item model (see Table 4).

**Table 3:** Fit statistics for confirmatory factor analysis of observations of hand preference

Model	*X* ^*2*^	df	*p*	RMSEA	CFI	TLI	SRMR/WRMR^a^
MLR							
7 items	19.69	14	0.14	0.05	0.96	0.94	0.08
WLSMV							
7 items	15.42	14	0.35	0.02	1.00	0.99	0.48

^a^SRMR is used for MLR estimation method, WRMR is used for WLSMV estimation method

**Table 4:** Model estimates for the seven handedness observations

Parameter	Unstandardized	Standard error	Standardized	Unstandardized	Standard error	Standardized
	MLR factor loadings			WLSMV factor loadings		
Maze	1.00^a^	–	0.77	1.00^a^	–	0.91
Ball	0.29	0.14	0.28	0.44	0.14	0.40
Draws	0.89	0.08	0.84	0.97	0.10	0.88
Fork	0.45	0.15	0.32	0.44	0.11	0.40
Knife	0.86	0.10	0.72	0.80	0.10	0.73
Toothbrush	0.43	0.15	0.29	0.49	0.12	0.44
Scissors	0.85	0.12	0.57	0.75	0.09	0.68
	MLR factor variance			WLSMV factor variance		
Handedness	1.21	0.26	1.00	0.82	0.11	1.00

^a^This parameter is used as the marker variable, fixed to a coefficient of 1.00, and so is not tested for statistical significance. All parameter estimates were statistically significant (*p* < 0.05)

Using these seven items, we created a composite handedness score ([RH-LH]/[RH + LH]*100). Handedness scores did not differ across LRC, HRA, and ASD children (H(2) = 0.77, *p* = 0.68). Most children had the early tendency to be right-handed or right-leaning (see Table 5).

**Table 5:** Categorical handedness groups across LRC, HRA, and ASD participants

	LRC	HRA	ASD	Total
Total *N*	69	60	22	151
Handedness category^a^ *N* (%)				
Right	51 (73.9%)	44 (73.3%)	14 (63.6%)	109 (72.2%)
Mixed, right-leaning	7 (10.1%)	9 (15.0%)	3 (13.6%)	19 (12.6%)
Mixed, no preference	4 (5.8%)	0 (0.0%)	2 (9.1%)	6 (4.0%)
Mixed, left-leaning	4 (5.8%)	2 (3.3%)	0 (0.0%)	6 (4.0%)
Left	3 (4.3%)	5 (8.3%)	3 (13.6%)	11 (7.3%)

^a^Handedness category based on calculated composite score (*C*) with common EHI categorical cutoffs: right (*C* > 40), mixed, right-leaning (0 > *C* ≥ 40), mixed, no preference (*C* = 0), mixed, left leaning (−40 ≤ *C* < 0), left-handed (*C* < −40)

### Associations between lateralization, handedness, and behavioral measures

Because there was no main effect of condition, we averaged the LSW laterality index across conditions and used this value in the following analyses. We found no significant relations between the LSW laterality index at 12 months and the four MSEL subscales at 12 months across all participants and within each of the groups (see Table 6). At 36 months, across all participants, there was a significant association between fine motor skills and handedness (*r*_*s*_ = 0.266, *p* = 0.001) such that children who had better fine motor skills were more likely to be right-handed. Within LRC children, the same association between fine motor skills at 36 months and handedness was found to be significant (*r*_*s*_ = 0.42, *p* = 0.000). Additionally, within the LRC children, a significant relation was found between expressive language abilities at 36 months and handedness (*r*_*s*_ = 0.37, *p* = 0.002). LRC children who had better expressive language abilities were more likely to be right-handed. No other associations between the four subscales of the MSEL and handedness at 36 months were significant (see Table 6).

**Table 6:** Correlations between MSEL subscales and LSW at 12 months and handedness at 36 months

		Group		
	All participants	LRC	HRA	ASD
	LSW laterality index at 12 months			
12-month MSEL *T* scores				
Fine Motor	0.03	0.11	0.05	−0.41
Visual Reception	−0.06	−0.37	0.23	−0.68
Receptive Language	−0.07	−0.34	0.10	−0.35
Expressive Language	−0.03	−0.04	−0.19	0.30
	Handedness score at 36 months			
36-month MSEL *T* scores				
Fine Motor	0.27**	0.42***	0.16	0.11
Visual Reception	0.03	0.08	0.04	−0.15
Receptive Language	0.16	0.25	0.14	−0.01
Expressive Language	0.18	0.37**	0.09	−0.02

***<0.001, **<0.01

**Fig. 4:**
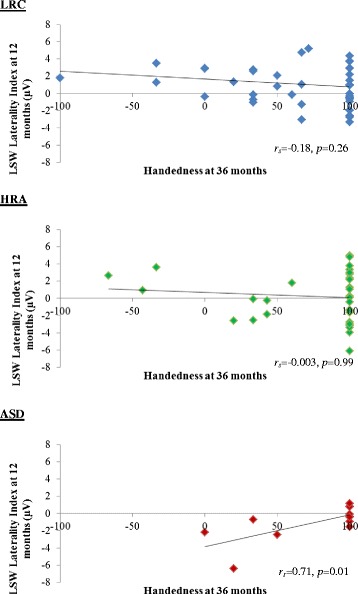
Association between LSW laterality index at 12 months and handedness at 36 months

LSW laterality index at 12 months did not significantly predict handedness at 36 months across all participants (*r*_*s*_ = −0.07, *p* = 0.51). Within each of the groups, LSW asymmetry did not predict 36-month handedness in LRC infants (*r*_*s*_ = −0.18, *p* = 0.259) or HRA infants *r*_*s*_ = −0.003, *p* = 0.988). However, for ASD children, there was a significant relation (*r*_*s*_ = 0.71, *p* = 0.010), such that children with a LSW laterality index more similar to LRC infants at 12 months were more likely to be right-handed at 36 months (see Fig. 4).

## Discussion

In this study, we examined early asymmetry patterns measured through neurophysiological responses to speech sounds and behavioral observations of handedness in children with and without ASD. By including both children with ASD and children who were at high familial risk for ASD, we could separate early ASD risk biomarkers from early ASD endophenotypes by comparing affected and unaffected family members. Analyses revealed group differences in their lateralization of ERPs to speech sounds at 12 months, but no group differences in handedness at 36 months. Handedness at 36 months was related to the children’s cognitive abilities, specifically fine motor skills and expressive language abilities within the LRC group. In children with ASD, we found an association between early lateralization patterns to speech and later handedness.

### Hemispheric lateralization

As we predicted, 12-month-olds who later developed ASD had reversed lateralization in the LSW in response to speech sounds compared to their typically developing peers. Moreover, this difference remained even in a subsample of infants without any language delays, suggesting that this finding is not simply a result of potential language difficulties within the ASD population. Similarly, we found no relations between this laterality index and cognitive abilities, including language abilities. Past work has found reversed lateralization in response to language in toddlers who were already exhibiting behavioral symptoms of ASD [67, 68]. Our current findings extend this by suggesting that atypical lateralization may be present as young as 12 months of age, before the overt symptoms of ASD emerge.

Our findings suggest that reversed lateralization to speech could serve as an early biomarker of risk for ASD, present before behavioral symptoms emerge. Importantly, we did not find reversed lateralization in the HRA group who did not go on to develop ASD, which suggests this is not an endophenotype. However, we cannot conclude that this atypical laterality to speech is specific in identifying children who go on to develop ASD. First, atypical asymmetry to language stimuli has also been found in children and adults with other complex neurodevelopmental disorders including schizophrenia [69] and specific language impairment [70, 71] and in two-month-old infants at risk for dyslexia [72]. Second, although not significantly different from either the LRC or ASD groups, HRA infants who did not go on to develop ASD within our sample showed an intermediate, dampened LSW laterality index with variability including values overlapping with the LRC and ASD range. Reversed lateralization by itself, therefore, might not be sensitive enough to identify which infants will go on to develop ASD from those who are simply at a familial risk for ASD or other neurodevelopmental disorders.

Given that reversed lateralization in response to speech might not be specific to children with ASD, a cumulative risk model with additional potential ASD biomarkers of risk might be better in identifying infants who will go on to develop ASD from those who will not [13, 73]. Other potential neural ASD biomarkers include various indices of reversed lateralization, such as atypicalities in structural lateralization patterns [74, 75] and reversed functional lateralization in domains outside of language, including face processing [76]. In fact, within our same sample, we have found atypical hemispheric lateralization in response to faces in HRA infants as young as 12 months. Moreover, preliminary evidence from this study found that infants who later received a diagnosis of ASD showed the most reversed lateralization within the HRA infants [20]. More work needs to be done to confirm this preliminary finding, but by understanding how these lateralization patterns manifest within the early development of ASD, we could increase the predictive power of early identification and explore the possibility of a larger underlying biomarker such as atypical development of cortical specialization.

### Handedness

Utilizing a novel observational approach, we investigated hand preferences in young preschoolers with and without ASD to create a constructed measure of handedness. Final analyses indicated an underlying unidimensional construct as the one-factor model with the following behaviors providing a good fit: hand used to draw, complete a picture-maze, cut with a knife, use a fork/spoon, cut with scissors, use a toothbrush, and throw a ball. Our findings are consistent with past work using the same analytic approach to demonstrate a more precise measure of handedness using the EHI in adults and adolescents [77, 78]. Interestingly, Dragovic’s [77] final revision of the EHI is almost identical to the measures included in our construct after he removed two items with large measurement error (i.e., hand used to open box with lid and upper hand when using a broom).

While these seven behaviors provide a measure of handedness in children, our models showed lower factor loadings compared to models of handedness in adults using similar behaviors [77, 79]. These lower factor loadings might represent the malleability of handedness in 3-year-olds. It is unclear when full, adult-like handedness emerges, but some researchers argue that the direction of handedness (left or right leaning) is fixed by 3 years and then continues to strengthen until late childhood with right-handed children showing stronger and earlier hand preferences than left-handed children [48]. Early inconsistencies in hand preference at age 3, therefore, could be reflected in the reduced factor loadings. Given these inconsistencies and the idea that adult-like handedness does not emerge until late childhood, using handedness as an early biomarker of ASD may be limited. Future work should follow the development of handedness from early toddlerhood into later childhood. Understanding the development of handedness, specifically emerging consistencies in hand preference, would lead to better understanding of potential differences in handedness across these groups.

Using these behaviors to create a handedness composite score, we found that the handedness measure did not differ across the groups at 36 months as most children showed a tendency to be right-handed or right-leaning. Given that older children with ASD have been found to exhibit less right handedness than their typically developing peers [55], the lack of a difference in our sample could be interpreted in several ways. First, our ASD sample showed stronger right-handed tendencies than past studies as our proportions of atypical handedness are on the lower end of the estimated ranges. In our sample, 22.7% of children with ASD were classified as mixed-handers while 13.6% were left-handers. Past studies investigating handedness in older children and adults with ASD have estimated higher proportions of incidences for both mixed- (17–47%) and left-handedness (18–57%; [38]). Our lower levels of left- and mixed-handedness might be a reflection of our sample’s characteristics. All but two of our children with ASD had average or above average cognitive abilities at 36 months as measured by the MSEL. While lower cognitive abilities are not present in all individuals with ASD, it has been estimated that as many as 55% of children with ASD have an intellectual disability [80]. Perhaps if our sample were more representative of the entire spectrum, we would find more variability in handedness given that individuals with intellectual disability are more likely to show left- and or mixed-handedness [81]. Second, our LRC children are only slightly right-handed biased. While the majority of LRC children were considered right-handed (73.9%), this proportion is lower than the estimated 90% in the general adult population [48]. The difference between groups in handedness, therefore, may emerge with age as the LRC children continue to strengthen their hand preference, particularly becoming stronger right-handers, relative to children with ASD.

Despite finding no differences on a group level, we did find an association between handedness and cognitive abilities. Specifically, across all participants the better the child was at fine motor skills at 36 months, the stronger their right-handedness. Closer inspection of this association indicated that this was driven by the LRC group. Most of the fine motor tasks require the child to be successful at activities involving one hand (e.g., drawing, placing pennies in a bank with a slot). Since young children, particularly left-handed children, often show inconsistencies in their hand preferences [48], this might be the result of a practice effect. Perhaps young children with stronger right-handed tendencies use their right-hand more in motor tasks requiring precision thus leading to better performance on these tasks. Additionally, we found that LRC children who were right-handed had stronger expressive language abilities. This is consistent with past work investigating this association in young typically developing populations [50, 51]. The lack of relation with handedness and cognitive abilities within the HRA and ASD population is surprising. However, perhaps this is, again, a reflection of our sample as the majority of these children had average or above average cognitive abilities. Future work should investigate the developing relation between handedness differences and cognitive abilities over a more representative sample.

### Behavioral and neural asymmetry

Across all participants, we did not find an association between speech lateralization at 12 months and handedness at 36 months. Looking within each group, although we found no relation in children without ASD, we did find a moderate association in children with ASD. Specifically, infants with ASD who exhibited a more typical lateralized response to speech at 12 months had stronger right-handed tendencies at 36 months.

On a group level, there is evidence of atypical cortical specialization in individuals with ASD with differences in structural lateralization [74, 75] and functional lateralization across many domains including face processing [76] and language [67, 82]. Moreover, atypical cerebral specialization in ASD is also reflected in the visible behavior of handedness as there are increased incidences of left- and mixed-handedness in children with ASD [55]. On an individual level, perhaps for the children with ASD in our sample, atypical lateralization to speech at 12 months and atypical handedness (left/mixed) at 36 months is a result of an underlying atypical cerebral specialization. Moreover, the children with ASD with more typical asymmetry patterns might have a protective factor in the early development of cortical lateralization which later manifests into more typical language lateralization and handedness.

Our findings of an association between early lateralization patterns and handedness in a small group of children with ASD as well as the lack of an association in children without ASD highlights many possibilities for future research including investigating different indexes of laterality and hand preferences. Specifically, by employing a longitudinal design and using a variety of neuroimaging methods, future studies could determine when atypicalities in structural hemispheric organization and functional lateralization in multiple domains, including face and language processing, first arise in low- and high-risk samples of children. On a behavioral level, observations of handedness and its consistency should be studied from infancy through childhood, starting with simpler actions in infancy such as grasping and manipulating objects and then moving to more complex adult-like behaviors such as writing. Comparing early lateralization patterns alongside with the growing stability of hand preferences would offer greater insight into how these might interact and lead to cortical specialization in individuals with ASD. Importantly, exploring individual differences within these patterns would potentially help in understanding heterogeneity of behaviors in ASD.

### Limitations

There are limitations to the current study that should be considered when interpreting the findings. One is the small sample size of the ASD group. A follow-up investigation with a larger number of infants would advance understanding about the early development of language and handedness within this population. Also, our study focused on a particular group of children who develop ASD: those at familial risk, and so it could be that atypical lateralization has a genetic or environmental underpinning and only runs in families with ASD. Future work should include other high-non-familial risk groups such as premature infants or infants who fail an ASD screener to test the generalizability of these findings. Another limitation is the absence of a reliable handedness measure to validate our novel observational approach for assessing handedness. While we did base our behaviors on an established handedness questionnaire, the EHI, we did not directly validate our measure against the EHI. Finally, we are unable to determine if the reversed lateralization to speech at 12 months is specific to ASD or whether this extends to other disorders. Future work should investigate infants at early risk for other neurodevelopmental disorders.

## Conclusions

To summarize: we found that, as a group, infants who were later diagnosed with ASD showed reversed lateralization of ERP response to speech at 12 months compared to HRA and LRC infants without ASD. Moreover, at the individual level, children with ASD with a more typical lateralization pattern at 12 months were more likely to show right-handed preferences at 36 months. Future work should continue to study the development of this relation as well as extend to other lateralization patterns, such as face perception, within children with ASD to determine if there is an underlying pattern of atypical cortical specialization. Such work will potentially help in uncovering early identifiers of ASD leading to earlier diagnosis and allowing for earlier interventions, optimizing outcomes for these individuals.

## Abbreviations

ADOS: Autism diagnostic observation schedule
ASD: Autism spectrum disorder
CFA: Confirmatory factor analysis
CFI: Comparative fit index
EEG: Electroencephalogram
EHI: Edinburgh handedness inventory
ERP: Event-related potential
HRA: High-risk for autism
LRC: Low-risk control
LSW: Late slow wave
MLR: Robust maximum likelihood
MSEL: Mullen Scales of Early Learning
RMSEA: Root mean square error of approximation
SRMR: Standardized root mean square residual
TLI: Tucker-Lewis index
WLSMV: Robust mean and variance adjusted weighted least squares
WRMR: Weighted root mean square residual

## Footnotes

1 Net type and amplifier were changed due to system upgrades that occurred midway through the project. We had no a priori reason to believe that the equipment changes would influence the data, and so they were not included as covariates in the analyses.
